# Biomechanics and Motion Analysis: From Human Performance to Clinical Practice

**DOI:** 10.3390/bioengineering12101114

**Published:** 2025-10-17

**Authors:** Chen He, Hong Fu, Christina Zong-Hao Ma

**Affiliations:** 1 School of Health Science and Engineering, University of Shanghai for Science and Technology, Shanghai 200093, China; hechen@usst.edu.cn; 2 Department of Mathematics and Information Technology, The Education University of Hong Kong, Hong Kong SAR 999077, China; hfu@eduhk.hk; 3 Department of Biomedical Engineering, The Hong Kong Polytechnic University, Hong Kong SAR 999077, China; 4 Research Institute for Smart Ageing, The Hong Kong Polytechnic University, Hong Kong SAR 999077, China

## 1. Introduction

Research in biomechanics and motion analysis quantifies motion, forces, and control strategies, bridging the gap between fundamental science and practical applications. By translating movement into measurable data, it enables performance optimization, injury prevention, and improved clinical outcomes. Recent technological advances have enhanced motion analysis, allowing for more precise and accessible assessment of human movement. Such progress has deepened our understanding of both normal and pathological motion patterns, directly informing the development of targeted interventions from sports training to rehabilitation therapies.

This Special Issue compiles nineteen original research articles and two review articles, examining three different research areas: (1) the biomechanics of performance, adaptation and injury risk in specialized populations; (2) the application of motion analysis in diagnosing movement disorders and guiding rehabilitation; and (3) innovations in measurement technologies that enhance accuracy, accessibility, and privacy. Together, these studies illustrate how biomechanical insights can inform evidence-based practice across sports, occupational, and clinical settings.

## 2. Contributions to This Special Issue

### 2.1. Biomechanics of Performance, Adaption and Injury Risk

Conducting motion analysis on professional populations who perform high-intensity tasks provides an evidence base for optimizing performance through individualized training strategies and reducing injury risk via targeted interventions. Regarding athletic populations, Fernández-Baeza et al. [1] used tensiomyography (TMG) to identify imbalances in quadricep muscles in professional football players, linking these asymmetries to injury risk and emphasizing the potential of targeted training to restore muscle balance. Similarly, Liu et al. [2] reported bilateral technical asymmetry among elite speed skaters during sprinting; although it did not hinder performance, this asymmetry could be perceived as an efficient compensatory strategy for high-speed motion. Furthermore, Zhou et al. [3] demonstrated that elite jump rope athletes adjusted to increasing tempos by reducing contact time and joint range of motion. This strategy for optimizing movement control offers practical guidance for improving training efficiency and stability.

Studying occupational participants, Kasović et al. [4] observed that carrying a standard 3.5 kg load significantly increased asymmetry in ground reaction forces and plantar pressures in police recruits, substantiating the associated musculoskeletal injury risks. Conversely, Rožac et al. [5] found that progressively increasing loads up to 45 kg did not significantly affect spatiotemporal gait asymmetry in experienced intervention police officers, indicating that long-term professional training may contribute to adaptive responses that preserve stability under load.

These adaptations are further nuanced by specific individual factors. Wang et al. [6] demonstrated that obstacle height amplified latent gender differences in joint biomechanics during crossing tasks. These findings reveal context-dependent motor control strategies and underscore the importance of considering individual biomechanical profiles in training programs.

### 2.2. Clinical Biomechanics of Motor Dysfunction and Rehabilitation

Conducting biomechanical analysis in a clinical setting facilitates the detection of subtle movement flaws and alterations that precede or accompany specific pathologies. Zhu et al. [7] compared reactive balance control methods in older adults who had a history of falling and those who did not, finding that the former tended to rely more on suspensory strategies to compensate for deficiencies in ankle and hip strategies, which led to longer recovery times. This provides a new perspective for identifying and implementing interventions for individuals at a high risk of falling. Chang et al. [8] noted significant kinematic alterations in adolescents after treatment for unilateral developmental dysplasia of the hip, suggesting that these changes may increase loading on the contralateral limb and the long-term risk of osteoarthritis. Similarly, Tovaruela-Carrión et al. [9] revealed that hallux limitus induces subtle but significant alterations in stance phase contact, emphasizing the value of early biomechanical detection in preventing functional decline.

Biomechanical analysis also drives the development of targeted, evidence-based treatments from wearables to surgical protocols. Wang et al. [10] demonstrated that wearable focal vibration therapy is a feasible and effective home-based intervention for improving key gait parameters in individuals with multiple sclerosis. Molteni et al. [11] developed and validated a novel upper limb kinematic protocol using a custom marker set (capturing three-plane movement plus trunk/head kinematics), which can be used in clinical applications for conditions such as cerebral palsy. In Jie et al.’s study [12], a new digitalized 3D spinal decompression and correction device (with longitudinal traction and multi-planar forces) was shown to significantly improve in-brace correction and comfort in adolescents with idiopathic scoliosis. A review by He et al. [13] confirmed that next-generation scoliosis braces are biomechanically comparable to traditional designs, but they require better ergonomic refinement and more researches into their long-term value in clinic. Ma et al. [14] analyzed lumbar stability after partial facetectomy, providing crucial biomechanical evidence for optimizing surgical approaches to preserve spinal integrity. Yan et al. [15] introduced an “assist-as-needed” control strategy for rehabilitation robots, enhancing active participation in patients with stroke by dynamically adjusting assistance based on their subjective intent.

### 2.3. Methodological Innovations in Motion Capture and Analysis

Reliable motion analysis requires continuous methodological refinement. In this Special Issue, several studies address the core technical challenges in this area. Yuhai et al. [16] introduced a deep learning-based method that significantly outperformed traditional approaches in recovering missing motion capture data, thereby enhancing data completeness. Lee et al. [17] demonstrated that machine learning can reliably differentiate genuine hemiplegic gait from mimicked patterns. Their model achieved good discrimination using ten key kinematic and force features, showcasing a pathway to efficient, data-driven clinical assessments. To guide the selection of motion capture systems, Rohrer et al. [18] compared “around-body” and “on-body” systems to provide evidence-based recommendations for choosing the appropriate technology for specific clinical conditions. In an ergonomic assessment, Lind et al. [19] revealed that the common ‘N-position’ can result in a substantial underestimation of arm elevation, questioning its validity without correction.

Regarding privacy concerns, Su et al. [20] presented a high-fidelity mmWave radar dataset for human pose estimation that inherently preserves privacy, addressing the critical need for monitoring in sensitive environments. Luttmer et al. [21] introduced a unique data library of human–object interactions that combines kinematic data with kinetic measurements of haptic forces, this is an important tool for researchers studying real-world behavior, bridging the fields of biomechanics, robotics, and virtual reality.

## 3. Conclusions

The studies in this Special Issue demonstrate how movement analysis can improve athletic training, occupational safety, and clinical rehabilitation. The integration of wearable sensors, artificial intelligence, and large-scale biomechanical data increases the precision and personalization of these applications. We hope to inspire continued efforts and collaborations to further advance the biomechanical analysis of human movement and its practical impact.
